# Reversal of Fetofetal Transfusion Syndrome in a Monochorionic Triamniotic Triplet: A Case Report

**DOI:** 10.7759/cureus.91269

**Published:** 2025-08-30

**Authors:** Erika Nakajima, Mio Taketazu, Saori Sugiyama, Kenrokuro Mitsube

**Affiliations:** 1 Obstetrics and Gynecology, Asahikawa-Kosei General Hospital, Asahikawa, JPN; 2 Pediatrics, Asahikawa-Kosei General Hospital, Asahikawa, JPN

**Keywords:** amniotic septostomy, fetal echocardiography, fetofetal transfusion syndrome, monochorionic triamniotic triplet pregnancy, reversal of fetofetal transfusion syndrome

## Abstract

In monochorionic multiple pregnancies, each fetus has its own amniotic sac but shares a single placenta. Along the placental surface, fetal vessels not only supply blood individually but also form anastomoses that cross between sacs, creating shared circulation. Such inter-fetal vascular connections can result in unequal blood volumes, a phenomenon termed "fetofetal transfusion" syndrome (FFTS). This imbalance is well-known in monochorionic diamniotic (MD) twins as twin‑twin transfusion syndrome, where the recipient fetus, subjected to volume overload, is at risk of heart failure, while the donor fetus experiences circulatory depletion. Diagnosis relies on discordant amniotic fluid levels: polyhydramnios in the recipient and oligohydramnios in the donor. Similar hemodynamic effects have been observed in monochorionic triamniotic (MT) triplet pregnancies. Reversal of FFTS is also well-documented in MD twin pregnancies; however, reports on a similar phenomenon occurring in MT triplet pregnancies are scarce in the literature. We encountered a case of FFTS in an MT triplet pregnancy in which it was suggested that reversal of FFTS occurred. At 19 weeks of gestation, one fetus (Fetus A) presented with polyhydramnios, whereas the other two fetuses (Fetuses B and C) exhibited oligohydramnios, fulfilling the criteria for FFTS. The patient experienced abdominal distension and cervical shortening, prompting amnioreduction. After serial procedures, an accidental amniotic septostomy between Fetuses A and C occurred as a procedural complication. Around the same time, the increase in amniotic fluid volume halted, and Fetus B, initially an oligohydramniotic fetus uninvolved in the septostomy, developed tricuspid regurgitation and right ventricular hypertrophy, along with increased amniotic fluid volume. Although this increase did not meet the criteria for polyhydramnios, which is required for FFTS diagnosis, the fetal echocardiographic findings and the postnatal symptoms of polyuria and hypertension suggested that reversal of FFTS may have occurred. A cesarean section was performed at 28 weeks of gestation due to Doppler abnormalities in Fetus C, and all three neonates were discharged without severe neurological sequelae. Following the septostomy, assessment of individual amniotic fluid volumes became infeasible, precluding definitive determination of donor-recipient status. However, the resolution of oligohydramnios in the initial donor Fetus B and the emergence of cardiac overload suggest the possibility of acute inter-fetal blood redistribution from either Fetus A or C. Although Fetus B did not meet the formal diagnostic criterion for polyhydramnios, postnatal symptoms support the hypothesis of reversal of FFTS. This case illustrates the potential for reversal of FFTS in MT triplet pregnancies and suggests that procedures such as amnioreduction or septostomy may serve as triggering events. Our findings also underscore the importance of fetal echocardiography in assessing donor-recipient status, particularly when traditional diagnostic criteria based on amniotic fluid volume become unreliable.

## Introduction

In monochorionic multiple pregnancies, each fetus has its own amniotic sac but shares a single placenta. In such pregnancies, some blood vessels running along the placental surface and connecting to the umbilical cords form vascular anastomoses, directly linking the two or three fetal circulations. These anastomoses can lead to an imbalanced blood flow among fetuses. Such inter-fetal vascular connections can result in unequal blood volumes, a phenomenon known as fetofetal transfusion syndrome (FFTS), which is well-documented in monochorionic diamniotic (MD) twins as the so-called twin-twin transfusion syndrome [1]. In this situation, the recipient fetus, subjected to volume overload, is at risk of heart failure, while the donor fetus experiences circulatory depletion. In MD twins, diagnosis relies on discordant amniotic fluid levels: polyhydramnios of ≥ 8 cm maximum vertical pocket (MVP) in the recipient and oligohydramnios of ≤ 2 cm MVP in the donor. Similar hemodynamic effects have been observed in monochorionic triamniotic (MT) triplet pregnancies [2].

In FFTS affecting MD twins, both fetuses are subjected to abnormal hemodynamic stress: hypovolemia in the donor and volume overload in the recipient. Additionally, the renin-angiotensin-aldosterone system is activated in the donor fetus in response to circulatory insufficiency. Excessively secreted hormones are then transferred to the recipient fetus, which is already suffering from volume overload, through vascular anastomoses in the placenta, creating a vicious cycle that exacerbates hypertension and cardiac dysfunction in the recipient fetus [3]. This results in cardiac hypertrophy, tricuspid regurgitation (TR), or, in severe cases, right ventricular outflow obstruction [3-5]. Characteristic echocardiographic findings or peripheral Doppler features reflecting this abnormal volume-pressure status can assist in evaluating the conditions of affected fetuses.

In MD twin pregnancies complicated by FFTS, a rare phenomenon known as "reversal of FFTS," in which the donor-recipient relationship is switched, has been reported [1,6-8]. Most documented cases involve reversal after fetoscopic laser photocoagulation (FLP) [6,7]; however, several cases without FLP have also been reported [6,7]. In such cases, a rapid reversal of hemodynamic status occurs between the fetuses, leading to abrupt clinical deterioration within a short period of one to three weeks. The reported perinatal mortality rate is 30-50%, which may exceed that of FFTS without reversal [1,6,7].

MT triplet pregnancies occur in approximately one in 100,000 births [9], with FFTS complicating 17% of cases [2]. In MT triplets, where vascular anastomoses may exist between each pair of fetuses, more complex hemodynamic alterations can occur than in MD twins. Although the reversal of FFTS is theoretically possible in MT triplets and, in some cases, following FLP has been reported [8], reversal of FFTS in MT triplets without FLP has been scarcely documented. The relative risk of adverse outcomes compared to twin pregnancies also remains unclear.

We presented a case of FFTS in an MT triplet pregnancy, in which reversal of FFTS may have occurred after amnioreductions complicated with an accidental amniotic septostomy. Although the diagnosis with amniotic fluid levels was impossible because of the amniotic septostomy, the fetal cardiac assessment helped evaluate the FFTS status.

## Case presentation

**Figure 1:**
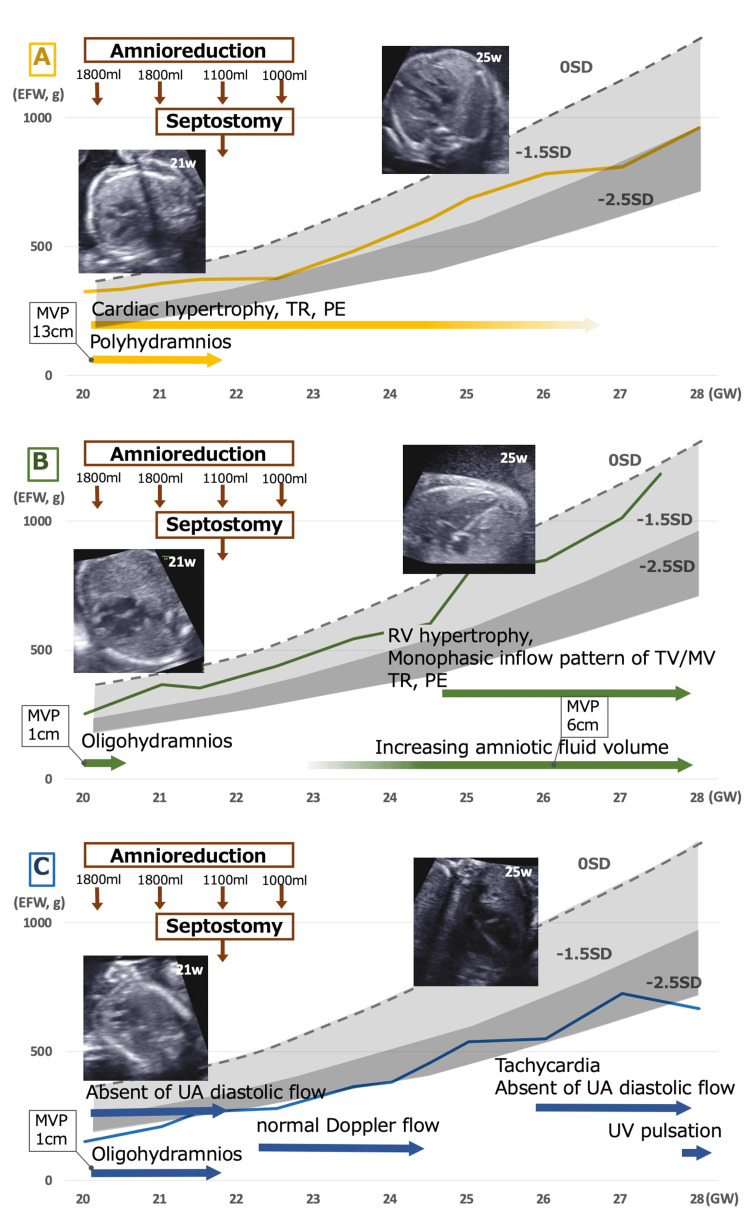
Trend of fetal growth and ultrasonographic findings of each triplet. Ultrasonographic findings include the estimated fetal growth, the amount of amniotic fluid, umbilical artery flow abnormality, and echocardiographic findings described above each bold arrow. The depicted graph compares the growth of each triplet (shown with a solid line) to the standard weight (indicated by a dashed line). The echocardiographic images in the figure depict the four-chamber views at 21 weeks and 25 weeks of gestation. (A) Fetus A. Polyhydramnios was observed before septostomy. Although findings for fetal cardiac overload remained, no significant deterioration was noted in the later course. The rate of increase in EFW decelerated, and the fetus eventually developed fetal growth restriction (FGR). (B) Fetus B. Findings indicative of fetal cardiac overload emerged at 24 weeks of gestation. (C) Although the assessment of the amniotic fluid volume was no longer feasible after septostomy, both fetal growth and Doppler flow patterns showed marked improvement immediately thereafter. EFW: estimated fetal weight, GW: weeks of gestation, TR: tricuspid valve regurgitation, PE: pericardiac effusion, RV: right ventricle, TV: tricuspid valve, MV: mitral valve, UA: umbilical artery, UV: umbilical vein, MVP: maximum vertical pocket

A 32-year-old primiparous woman became pregnant after a single blastocyst transfer and was diagnosed with an MT triplet pregnancy. She was admitted to our hospital at 19 weeks of gestation and was diagnosed with FFTS because one fetus (Fetus A) presented with polyhydramnios (MVP of 13.0 cm) and the other two fetuses (Fetuses B and C) presented with oligohydramnios (MVP of 1.9 cm each). Fetus A’s heart wall was thick (Figure 1), and the thicknesses of the right ventricular wall, the interventricular septum, and the left ventricular posterior wall were 2.9 mm (Z score (Z) = 3.8), 4.1 mm (Z = 11.7), and 3.4 mm (Z = 4.6) [10] each in 21 weeks of gestation. TR and pericardial effusion were also observed, suggesting cardiac pressure and volume overload. Fetus A’s Doppler inflow waveforms of the tricuspid and mitral valve exhibited a monophasic pattern. Fetus B showed normal Doppler flow patterns. Fetus C’s umbilical artery (UA) diastolic flow was absent. Fetuses A, B, and C’s estimated fetal weights were 326 g (SD: 0.7), 253 g (SD: -0.9), and 151 g (SD: -3.0), respectively.

The pregnant woman complained of severe abdominal distension. She also exhibited uterine cervical shortening symptoms, presumed to be caused by increased intrauterine pressure. Although FLP was considered, given its growing use in FFTS among MT triplets [2], her condition precluded referral to a facility offering FLP. Therefore, amnioreduction was planned to alleviate symptoms and prevent preterm labor. The procedures were conducted through the amniotic sac of Fetus A, which exhibited polyhydramnios in 19 weeks of gestation. Fetus A’s amniotic fluid volume re-increased after the first and the second amnioreductions, and it became polyhydramnios again so that the third amnioreduction was needed (Figure 1). At the third puncture, the needle unintentionally perforated an amniotic membrane between Fetus A and Fetus C as a procedural complication. As a result, the amniotic fluid volumes of Fetus A and Fetus C could no longer be assessed separately, rendering further evaluation of FFTS progression impossible based on the discordance of amniotic volume. However, the size of the A-C common cavity stopped increasing after the third puncture, and it began to decrease from the fourth puncture onward. The UA Doppler flow of the initial donor Fetus C returned to normal four days after amniotic septostomy, and fetal growth gradually resumed (Figure 1). The growth of the initial recipient, Fetus A, gradually slowed, and by 27 weeks of gestation, the estimated fetal weight had declined to below -1.7 SD. The echocardiographic findings of cardiac overload gradually resolved.

The amniotic fluid volume of the initial donor Fetus B increased to within the normal range three days after the first amnioreduction. It contributed to a gradual rise, reaching an MVP of 6 cm at 26 weeks of gestation. In addition, Fetus B presented with TR and right ventricular hypertrophy with a wall thickness of 3.6 mm (Z = 3.8) at 25 weeks of gestation. As no structural cardiac anomalies leading to the right heart abnormalities, such as tricuspid dysplasia or restrictive arterial duct, were seen, the findings suggested intrauterine volume and pressure overload. Despite not meeting polyhydramnios criteria, the weight gain and cardiac findings raised suspicion that Fetus B became the new recipient.

Uterine symptoms improved after the fourth amnioreduction, and no further procedures were needed.

Finally, Fetus C showed growth arrest again, and a cesarean section was performed at 28 weeks of gestation because the umbilical venous pulsation appeared in Fetus C. Three female babies were born, with their birth weights of 834 g (SD: -1.6) for Fetus A, 1,066 g (SD: -0.4) for Fetus B, and 626 g (SD: -3.3) for Fetus C. Fetus B, the heaviest, presented with neonatal hypertension, myocardial hypertrophy, and polyuria without structural anomalies, confirming its status as the probable recipient. All three infants had normal karyotypes, were fed orally, discharged at four months, and exhibited normal neurodevelopment at five years of age.

**Figure 2:**
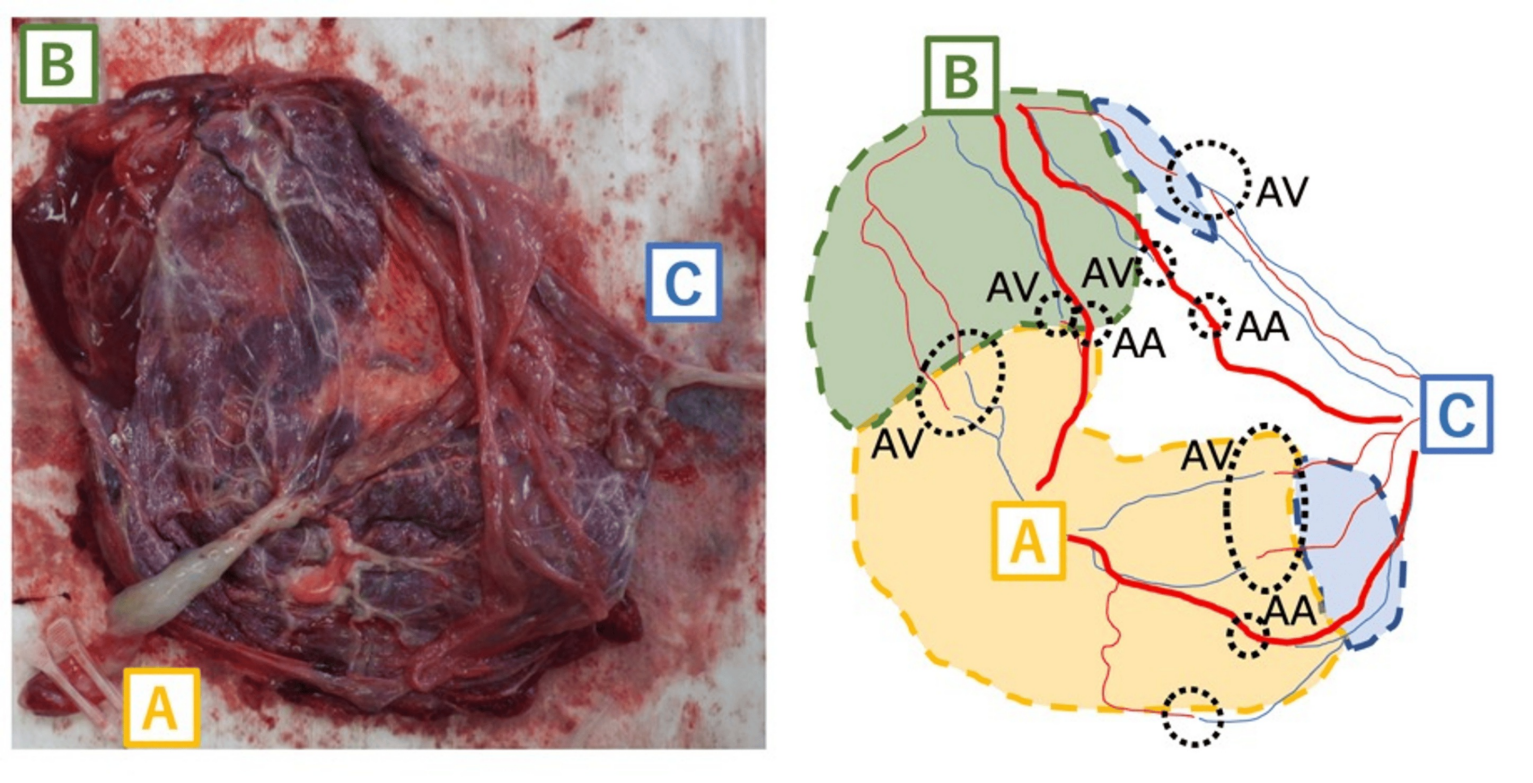
The findings and schema of the placenta. The territory of Fetus C was extremely small, and its umbilical vessels were inserted on the placental margin. Each pair of the three fetuses had several arterial-artery or arterial-venous anastomoses. Image Credits: Nakajima (author) AA: arterial-artery anastomoses, AV: arterial-venous anastomoses

In placental pathology, Fetus C’s area was smaller than 10%, and most of her placental blood vessels were within the membrane (Figure 2). There are many complicated arterial-artery (AA) or arterial-venous (AV) anastomoses among every pair of fetuses.

## Discussion

Reversal of FFTS has been most frequently reported after FLP, and the presumed mechanism involves residual vascular anastomoses left unsealed during the procedure, allowing sudden hemodynamic reversal [7,8]. Non-laser reversal cases, though rare, have occurred following amnioreduction or spontaneously [6,7]. Some reports suggest that decompression of the amniotic cavity during amnioreduction may alter placental vessel compression, triggering abrupt hemodynamic shifts via anastomotic pathways [11,12]. While the reversal of FFTS in MT triplet pregnancies has not been documented, it could potentially manifest through more complex mechanisms, such as involvement of a third fetus or larger inter-fetal blood shifts, compared to twin pregnancies.

In observational studies of MD twin pregnancies complicated by FFTS, many recipient twins develop cardiomyopathy secondary to chronic volume overload, while donor twins often exhibit Doppler flow abnormalities reflective of circulatory insufficiency. Notably, up to 70% of recipient fetuses display echocardiographic evidence of structural or functional cardiac compromise, including ventricular hypertrophy, cardiomegaly, diastolic dysfunction, and atrioventricular valve regurgitation [4,5,13].

In this case, Fetus A initially presented with features of a recipient; the largest weight, polyhydramnios, myocardial hypertrophy, and TR, which resolved after amnioreduction and septostomy. Fetus C, initially a donor, showed transient improvement but was ultimately born with donor-like features (Figure 1). After the septostomy, it becomes difficult to discriminate between each fetus’s status as a donor or a recipient based on the amniotic fluid volume. However, the resolution of cardiac overload in Fetus A suggests that it was no longer the recipient. Fetus B was initially considered a donor. Although the amniotic sac of Fetus B was not involved in the septostomy, the amniotic fluid volume increased, fetal weight gained, and cardiac signs of right heart volume and pressure overload appeared after the amnioreduction. Postnatally, Fetus B continued to show evidence of cardiac overload and polyuria. Although the amniotic fluid volume did not meet the criteria for a recipient, these findings raise the possibility that Fetus B functionally became the recipient.

As described above, the series of changes observed after septostomy did not allow for a definitive diagnosis of FFTS based on amniotic fluid discordance in the present case; thus, it is not possible to assert with certainty that true reversal of FFTS occurred. However, given the absence of other plausible causes, alterations in fetal growth patterns, together with echocardiographic findings, may reasonably indicate the occurrence of reversal of FFTS.

Previous studies have suggested that amnioreduction can cause FFTS reversal via placental decompression and blood redistribution through anastomoses [11,12]. However, all of these reports describe this phenomenon only in twin pregnancies, where a closed circulation is established between two fetuses through shared vascular anastomoses. In the placenta of this case, both AA and AV anastomoses were identified between each pair of fetuses. If such a reversal were to occur in an MT triplet pregnancy, the hemodynamic exchange between two fetuses could potentially be modulated or even amplified by vascular anastomoses involving the third fetus. Given this complexity, it is difficult to predict or fully explain the hemodynamic changes based on this single case. Future similar case reports may help elucidate these mechanisms.

Additionally, this case highlights the potential utility of fetal echocardiography for monitoring inter-fetal hemodynamic changes in MT triplets when amniotic fluid assessment is limited. As the observation points in fetal follow-up and post-natal management are completely different between the recipient and donor in FFTS, the estimation of fetal status in FFTS is crucially important. In the future, targeted monitoring strategies, such as serial fetal echocardiography in multidisciplinary surveillance protocols, may help validate the role of these tools in the diagnosis and management of FFTS in triplet pregnancies.

## Conclusions

Herein, we presented a rare case of FFTS in an MT triplet pregnancy in which reversal FFTS was suspected. The observation of significant hemodynamic changes following the initial development of FFTS suggests that phenomena similar to reversal FFTS in MD twin pregnancies may also occur in MT triplets. As no vascular anastomoses were disrupted by FLP, further investigation is warranted to elucidate what factors may trigger such hemodynamic alterations.

Additionally, this case suggests that fetal echocardiography, in addition to amniotic fluid volume assessment, may be a useful tool for monitoring inter-fetal circulatory changes in FFTS among MT triplets.
